# Genetic Diversity and Virulence Profiling of Multi-Drug Resistant *Escherichia coli* of Human, Animal, and Environmental Origins

**DOI:** 10.3390/antibiotics11081061

**Published:** 2022-08-04

**Authors:** Asfand Yar, Muhammad Adil Choudary, Abdul Rehman, Abid Hussain, Amina Elahi, Farooq ur Rehman, Ahmed Bilal Waqar, Abdulrahman Alshammari, Metab Alharbi, Muhammad Atif Nisar, Mohsin Khurshid, Zaman Khan

**Affiliations:** 1 University Institute of Medical Laboratory Technology, Faculty of Allied Health Sciences, The University of Lahore, Lahore 54000, Pakistan; 2 Central Park Medical College, Lahore 54000, Pakistan; 3 Institute of Microbiology and Molecular Genetics, University of the Punjab, Lahore 54000, Pakistan; 4 Department of Microbiology and Molecular Genetics, Faculty of Life Sciences, University of Okara, Okara 56300, Pakistan; 5 Department of Pharmacology and Toxicology, College of Pharmacy, King Saud University, P.O. Box 2455, Riyadh 11451, Saudi Arabia; 6 College of Science and Engineering, Flinders University, Adelaide 5042, Australia; 7 Department of Microbiology, Government College University, Faisalabad 38000, Pakistan

**Keywords:** *Escherichia coli*, ERIC-PCR, virulence genes, biofilm producers

## Abstract

Rapid urbanization has increased human-animal interaction and consequently enhanced the chances to acquire zoonotic diseases. The current investigation is focused to uncover the genetic diversity of multidrug-resistant *E. coli* strains between different ecologies (i.e., humans, livestock, and environment) at the molecular level by employing antimicrobial resistance profiling, virulence genes profiling, and microbial typing approach using ERIC PCR. Based on multiple antibiotic resistance, overall, 19 antibiotic resistance patterns (R1–R19) were observed. Most of the strains (49/60) were detected to have the combinations of *stx*, *eaeA*, and *hlyA* genes and considered STEC/EPEC/EHEC. A total of 18 unique genetic profiles were identified based on ERIC-PCR fingerprints and most of the strains (13) belong to P1 whereas the least number of strains were showing profiles P7 and P8-P11 (one member each profile). The calculated values for Shannon index (H) for human, animal, and environment are 1.70, 1.82, and 1.78, respectively revealing the highest genetic diversity among the *E. coli* strains of animal origin. The study revealed that drug-resistant pathogenic *E. coli* strains could be transmitted bidirectionally among the environment, humans, and animals.

## 1. Introduction

The emergence and re-emergence of infectious diseases are one of the biggest challenges being faced by humanity [1]. Nearly 60% of infectious and 75% of emerging diseases are zoonotic in origin and could be transmissible to humans due to a shared environment [2,3]. Around 30% of the land surface is occupied by livestock settings with a significant global asset worth $1.4 trillion contributing 33% to agricultural gross domestic production (AGDP). In Pakistan, the livestock sector contributes 11% to AGDP and 13.4% to overall GDP and engages 35 million people [4]. The infectious diseases among the livestock pose a significant burden on the country’s economy. Moreover, it increases the chance of the disease transmission to humans via environmental route [5].

Human health is affected in a variety of ways by the environment, either directly via human exposure to harmful agents (physical, chemical, and biological) or indirectly due to disrupted ecosystems. World Health Organization (WHO) has estimated that 13 million deaths per annum are attributable to preventable environmental causes [6]. The burden of environmental diseases is 15 times greater in developing countries as compared to developed ones, due to differences in contact with environmental risks and approaches to health care [7]. One health approach illustrates that the health of people is dependent on and interconnected to animal and environmental health [8]. Over the past decade, the concept of one health is widely being accepted in the developing as well as the developed world. The professionals from the clinical, veterinary, and environmental sectors should work jointly in a coordinated manner using one health approach for the amelioration of the increasing burden of infectious diseases [9].

Antibiotics are helpful as a therapeutic agent for the treatment of infectious diseases in humans. Further, antibiotics are also used broadly in the aquaculture and livestock industry [10]. Consequently, antimicrobial resistance is developing among bacteria in clinical and veterinary settings, which can spread bacteria in humans, livestock, and environmental sectors either due to the movement of bacteria in different ecologies or transmission of resistant determinants that can be facilitated by mobile genetic elements [11]. Antibiotic resistance in Pakistan is increasing because of misuse and/or overuse of antibiotics in humans, animals, and agricultural practices [12].

This study was designed to assemble the information regarding the antibiotic resistance in *E. coli* strains from animals, humans, and the environmental settings of Pakistan and to uncover the underlying molecular mechanism of pathogenesis. Moreover, the genetic diversity of strains isolated from all these environments was assessed by ERIC-PCR.

## 2. Results

A Total of 60 non-duplicated *E. coli* strains were recovered from environmental sources (wastewater *n* = 13 and soil *n* = 7), animal origin (cattle feces *n* = 9, intestines *n* = 6, and milk *n* = 5), and human origin (urine *n* = 6, pus *n* = 5, sputum *n* = 5, blood culture *n* = 2 and tissue *n* = 2).

### 2.1. Antibiotic Resistance Profiling

Overall, out of a total of 60 *E. coli* strains, the highest resistance was shown against ampicillin and ceftazidime followed by gentamicin, cefotaxime, ciprofloxacin, levofloxacin, and tigecycline (Table S1). Furthermore, five out of 20 human isolates and two out of 20 animal isolates were XDR, whereas no *E. coli* strain isolated from the environment was XDR.

Based on multi-drug resistance, overall, 19 antibiotic resistance patterns (R1–R19) were observed (Table 1). Among them, the R6–R10 pattern was unique to *E. coli* of human origin, patterns R3–R5 and R17–R19 were unique to the environment, and pattern R13 was unique to animal isolates. On the other hand, patterns R1 and R11 were found among all three sources of *E. coli*, while patterns R2 and R6 were found in human and environmental niches and patterns R12 and R14–R16 were found to be shared between *E. coli* of animal and environmental origin. Overall R12 was the more prevalent resistance pattern, while R2, R11, and R12 were more prevalent in human, animal, and environmental isolates, respectively.

Furthermore, only two profiles (R2 and R6) were common between humans and the environment whereas four profiles (R12, R14, R15A, and R16) were found common between animals and the environment indicating more close interaction between animals with the environment.

Cluster analysis of *E. coli* strains from human, animal, and environmental origins based on antimicrobial resistance patterns towards nine antibiotic groups was performed to show the similarity among strains from different origins as well as to exhibit the richness of ecological niches. Cluster analysis of *E. coli* strains of human origin revealed two distinct clusters (A and B) consisting of 19 members and one single member cluster mainly based on the susceptibility to monobactams and macrolides with 20% to 100% similarity (Figure S1a). Cluster A has six subclusters and consists of 16 strains out of which five are XDR and 11 are MDR. Four XDR strains share the same locality i.e., sputum and one recovered from blood culture. All the XDR strains are constituting the same subcluster with 100% similarity. Strains recovered from urine (U2, U3, and U4), pus (P1–P4), and tissue (T1) constitute the same spectrum with 100% similarity. Strains U6 and P5 are showing distinct resistance profiles. Cluster B comprises three strains from different localities (urine, tissue, and blood cultures) with 35% to 100% similarities.

Cluster analysis of animal origin strains revealed two major distinct clusters (A and B) and four subclusters with 16.7% to 100% similarity (Figure S1b). Cluster A is mainly based on the susceptibility to monobactams while cluster B is based on the resistance against penicillin and cephalosporins. Cluster A includes two XDR (from cattle feces and the intestine) and one MDR strain with 65% to 100% similarity. Cluster B is larger with four subclusters consisting of 17 MDR strains. Most of the strains are showing a homogenous resistance pattern and are included in the same subcluster.

Environmental strains have exhibited the most diverse and richest resistance patterns with 10% to 100% similarity. There are two major clusters (A and B) along with 14 subclusters (Figure S1c). Cluster A is comprised of one single member subcluster bearing one XDR strain (from soil) and one double-members subcluster of two MDR strains (one from soil and one from wastewater), with 40% to 100% similarity. Cluster A is mainly based on the resistance against penicillins, cephalosporins, and fluoroquinolones. Cluster B is the larger cluster grouped with 17 MDR strains with 10% to 100% similarity and is primarily based on the resistance against penicillins and cephalosporins. Soil strains (ES1, ES2, and ES5) are groups in the same subcluster with 80% to 100% similarity while wastewaters strains (W5, W7, W8, W9, and W11) are included in the same subcluster with 100% similarity with each other and 80% similarity with other strains of the large cluster. Wastewater strains W1, W4, and W13 are constituting single member subclusters showing their uniqueness while W10 and W12 are grouped in a subcluster.

**Figure 1:**
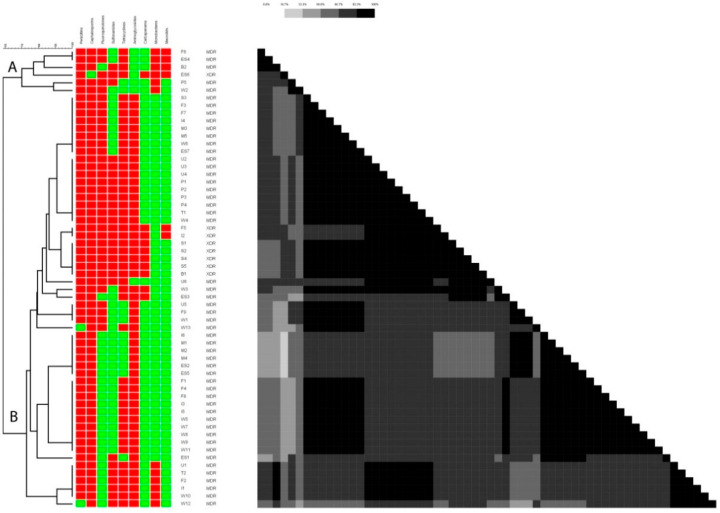
Overall clustering of *E. coli* strains from humans, animals, and the environment is showing similarities across different origins. The red color is representing resistance while the green color is indicative of susceptibility to the antibiotic mentioned in the same lane.

Overall clustering of all the *E. coli* strains from human, animal, and environmental origins were done to detect their hierarchical relatedness based on antibiotic resistance patterns (Figure 1). There are two large distinct clusters (A and B) with 22 small subclusters which are 10% to 100% similar. Cluster A is a relatively small cluster with only six strains while cluster B consists of 54 strains.

Different pathotypes of *E. coli* were identified based on virulence genes and their combinations. The strains carrying *stx*1 and/or *stx*2 genes are declared as STEC, the strains bearing *eaeA* gene are considered EPEC, the strains harboring the *hlyA* gene are declared as EHEC and the strains showing combinations of these virulence genes were typed accordingly. There were only 5/60 strains that carry *eaeA* gene alone and were declared as EPEC and the 1/60 strain was identified as EHEC as it harbored the *hlyA* gene, whereas all other strains were found to harbor more than one virulence gene (Table 2). Most of the strains were detected to have the combinations of *stx*, *eaeA*, and *hlyA* genes and considered STEC/EPEC/EHEC. Moreover, 2/60 strains were identified as EPEC/EHEC, 2/60 as STEC/EHEC, and one as STEC/EPEC (Figure S2). The difference in the distribution of these pathotypes among the three ecologies is found statistically significant with a *p*-value of 0.019. It is noticeable that human origin *E. coli* strains are showing all the mentioned pathotypes while animal and environmental origin strains are showing only two of the pathotypes. All the uropathogenic strains and strains recovered from cattle feces, cattle milk, and soil were found to belong to pathotype STEC/EPEC/EHEC which shows the firm interaction of all these ecologies (Figure S3). Moreover, it is observable that there are 18 strains of animal and environmental origin each (total of 36) and 13 human origin strains that carry the combination of all the studied genes (*stx*, *eaeA*, and *hlyA*) indicating the abundance of these genes as well as greater opportunities of transferring the genes between the strains in different niches.

Valuable information has been extracted by comparing *E. coli* pathotypes with antibiotic resistance profiles i.e., STEC/EPEC/EHEC pathotypes are showing all the resistance profiles except R8 and R10. EHEC, EPEC/EHEC, and STEC/EPEC pathotypes are found to be resistant to fewer antimicrobials and show only one profile each while EPEC and STEC/EHEC are exhibiting five and two profiles, respectively (Figure S4). These results suggest the involvement of virulence genes in the emergence of antimicrobial resistance.

### 2.2. In-Vitro Biofilm Production

The pathogenic potential of bacteria is found to increase when they acquire biofilm formation capabilities in addition to the presence of virulence genes. Overall, 48/60 *E. coli* isolates were able to form biofilm even in the absence of an environmental stressor. Among 48 isolates, 13 were strong, 18 were moderate and 17 were weak biofilm producers (Table S2). Moreover, the resistance and sensitivity of biofilm producer and non-producer strains to various antimicrobial groups were evaluated (Table 3). It was observed that 47/48 biofilm producer strains were resistant to penicillins and cephalosporins whereas 11/12 and 12/12 (100%) non-producer strains were resistant to both these groups, respectively. Macrolides were found the most effective drugs for biofilm producers and carbapenems from non-producers as only 10% of producer strains were able to resist macrolides and all the non-producer strains were susceptible to carbapenems. It was very evident from the resistance susceptibility pattern of producers and non-producer strains that producer strains resisted more antibiotics to a greater extent attributed to their biofilm-producing capabilities.

### 2.3. ERIC-PCR Based Fingerprinting

**Figure 2:**
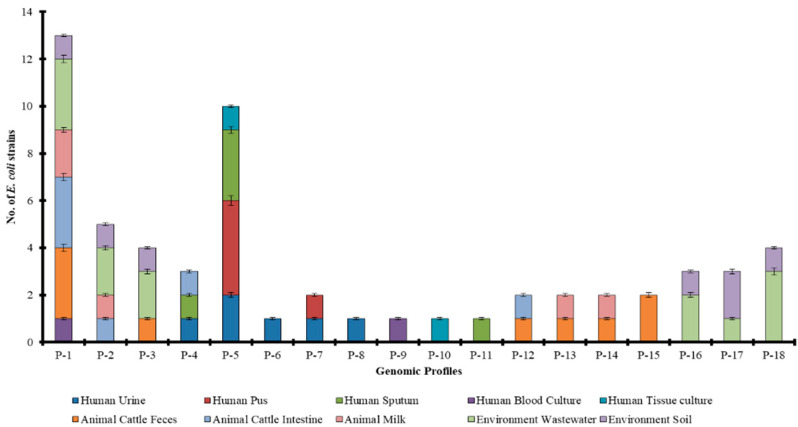
Dissemination pattern of genetic profiles among human, animal, and environmental origins and sources.

ERIC-PCR results showed that in all the 60 strains, 20 distinct bands were observed (Table S5). The maximum number of the bands was seven and the minimum was one that appeared in a single strain. The strains from each source were divided into different profiles based on banding patterns. A total of 18 unique genomic profiles were identified based on ERIC-PCR fingerprints in 60 *E. coli* strains from human, animal, and environmental sources (Figure 2). Most of the strains (13) belong to P1 whereas the least number of strains are showing profiles P7 and P8–P11 (one member in each profile). Profile P1 is shared among strains from all origins, while profiling P2 and P3 are shared among animal and environmental strains, and profile P4 is shared among human and animal origin strains. Moreover, genomic profiles P5–P11, profiles P12–P15, and profiles P16–P17 are restricted to the human, animal, and environmental strains, respectively. Profile P1 is prevalent among *E. coli* of both human and animal origins whereas profiles P1 and P18 are equally common in environmental strains. This differential distribution of genomic profiles in human, animal, and environmental ecologies is found statistically significant (*p*-value = 0.011).

### 2.4. Source Wise Genetic Diversity

Overall, 18 unique ERIC-PCR fingerprints were obtained from 60 *E. coli* strains of various origins. The calculated values for Shannon index (H) for human, animal, and environment are 1.70, 1.82, and 1.78, respectively revealing the highest genetic diversity of *E. coli* strains of animal origin followed by environment and human origins. In humans, sputum is observed as the source of most diverse *E. coli* (H = 0.95) followed by blood culture (H = 0.69), tissue (H = 0.69), and urine (H = 1.56) and pus (0.50). Moreover, in animal-origin *E. coli* strains, the genetic diversity is found highest for the *E. coli* population of cattle feces (H = 1.67), followed by cattle milk (H = 1.33) and cattle intestine (H = 1.24). The Shannon diversity index values for soil and wastewater sources are 1.748 and 1.738, respectively. These results declare soil followed by wastewater as the most diversified niches of the *E. coli* population (Table 4).

The values for the Simpson diversity index (1-D) for the human, animal and environmental origin *E. coli* are 0.71, 0.78, and 0.83, respectively which is also indicating that the environmental niche is harboring a more genetically diverse *E. coli* population (Table 4). The values for the evenness (E) index for human, animal, and environment are 0.61, 0.78, and 0.99, respectively demonstrating that *E. coli* strains are more evenly distributed in the environmental niche as compared to animals and humans. Furthermore, the richest diversity was found in *E. coli* from humans with nine unique genomic profiles followed by animals (eight profiles) and environment (six profiles). The soil, wastewater, and cattle feces are the richest sources with six unique profiles each followed by urine (five profiles), cattle intestine (four profiles), cattle milk (four profiles), sputum (three profiles), tissue (three profiles) and blood (three profiles).

### 2.5. ERIC-PCR-Based Clustering

**Figure 3:**
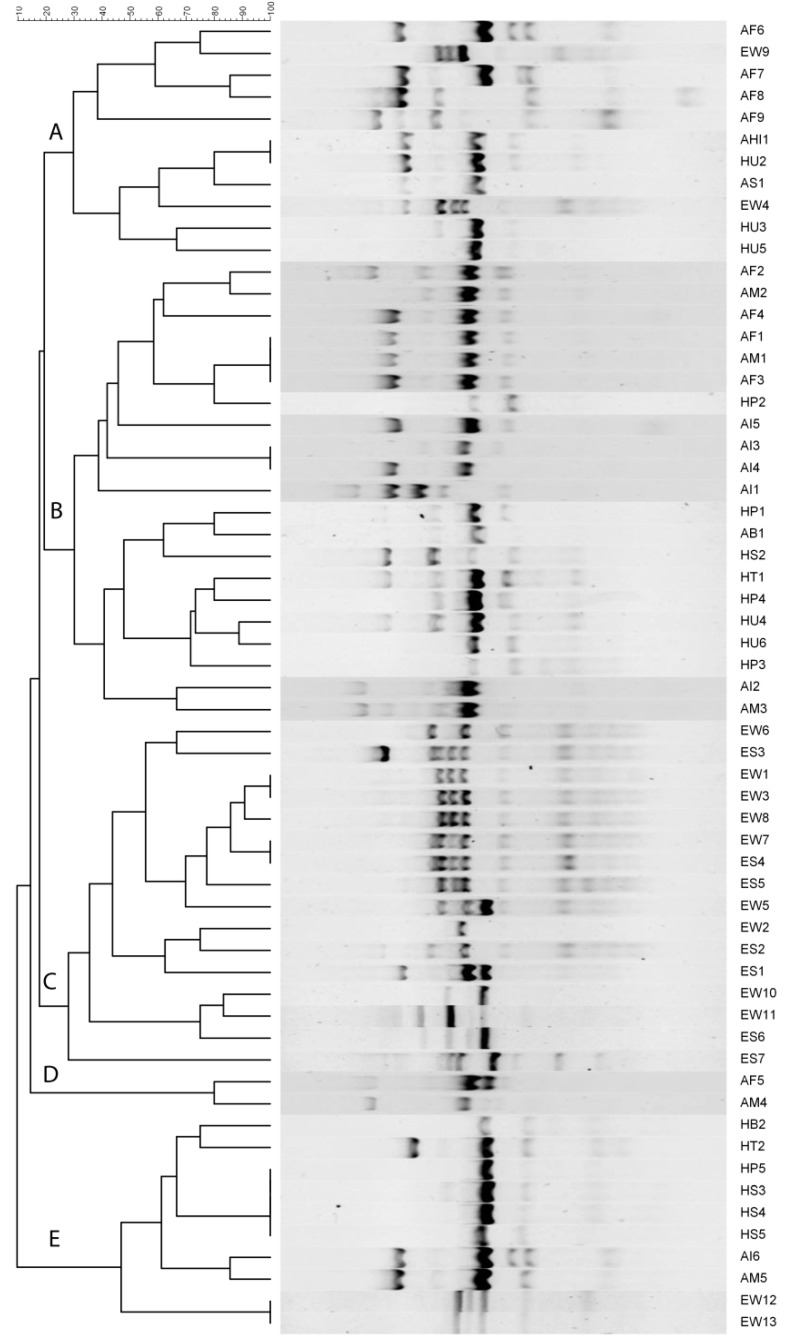
Combined clustering of all the *E. coli* strains from human, animal, and environmental origins based on ERIC-PCR fingerprinting patterns.

Dendrograms were constructed for the human, animal, and environmental strains independently (Figure S5) as well as a combined dendrogram showing genetic similarity and diversity among strains from different ecologies (Figure 3). The detail of ERIC-based fingerprints of genetic profiles has been summarized in Table S3. Human strains showed two distinct clusters (A and B) at about a 35% similarity level. Cluster B is smaller and groups only seven strains at 70% similarity while Cluster A is including 13 strains in four subclusters at 65% similarity. Five strains (HP1, HP2, HU4, HT1, and HP3) are showing the same banding pattern with 100% similarity (Figure S5a). Animal strains have also been found arranged in two large distinct clusters at 20% similarity. Cluster A can be divided into two subclusters at 80% similarity while cluster B is shown to have three subclusters at 80% similarity (Figure S5b). Environmental strains have shown the highest genetic diversity with five distinct clusters at 40% similarity and four subclusters at 70% similarity (Figure S5c). The combined dendrogram (Figure 3) is showing the 60 strains clustering into 15 large clusters at 35–40% similarity and 15 subclusters at 60% similarity.

**Figure 4:**
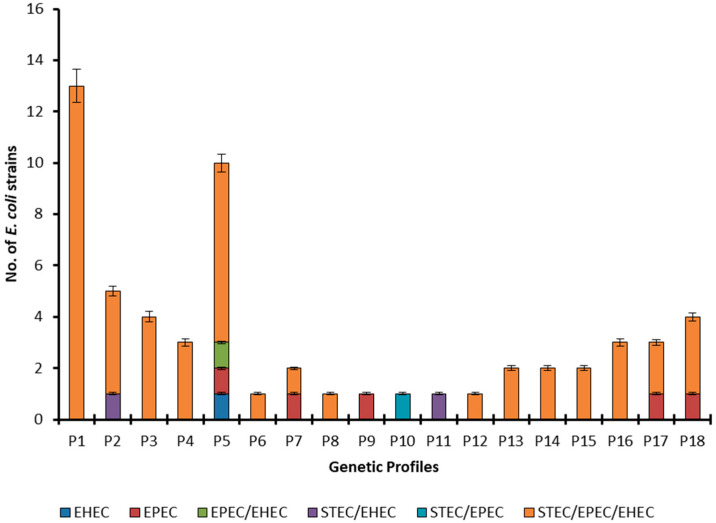
ERIC-PCR-based fingerprint patterns of *E. coli* pathotypes.

To the best of our knowledge, it is the first study from this part of the world that is reporting the fingerprints of various *E. coli* pathotypes from human, animal, and environmental origins. The pathotype STEC/EPEC/EHEC has been observed genetically more diverse and shows all the genetic profiles except profiles P9, P10, and P11 (Figure 4). Most of the members of this pathotype belong to profile P1 followed by P5. The profiles P2 and P3 are shown by four members each, profiles P4, P16, and P18 by three members each, profiles P13, P14, P15, and P17 by two members each, and profiles P6, P7, P8, and P12 by one member each. Moreover, only one strain was detected with EHEC pathotype and it belongs to genetic profile P5, while all the EPEC strains belong to different genetic profiles. The pathotypes EPEC/EHEC and STEC/EPEC have one member each and these belong to profiles P5 and P10, respectively. The pathotype STEC/EPEC is found to show two types of fingerprints (P2 and P11).

**Figure 5:**
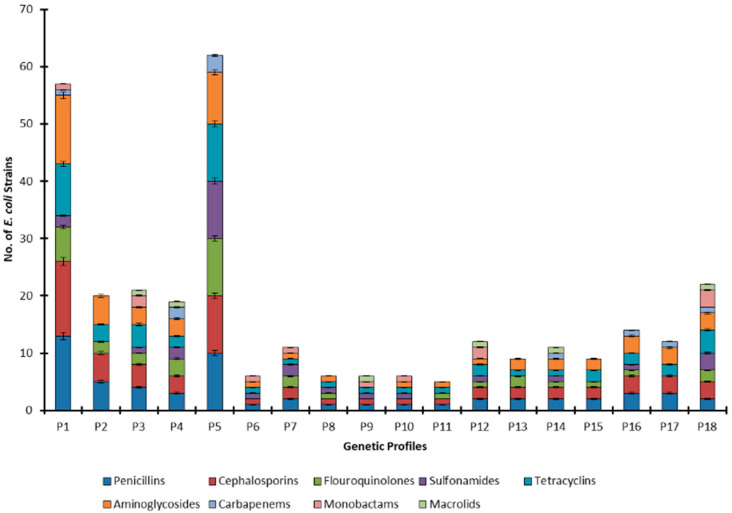
Antibiotic resistance pattern of *E. coli* strains based on genetic profiles.

Valuable information was extracted by cross tabulating the genetic profiles and antibiotics resistance and found that the strains exhibiting genetic profile P1 were resistant to all the tested antibiotics except macrolides and the strains belonging to profile P18 were resistant to all the tested antibiotic groups. Penicillins and cephalosporins have been proved least effective while macrolides followed by carbapenems and monobactams are most effective against the genetically diverse *E. coli* strains (Figure 5).

## 3. Discussion

The emergence of antibiotic resistance in *E. coli* is of great concern as the infections caused by resistant bacterial pathogens are difficult to manage. The current investigation is revealing that antibiotic resistance is not restricted to *E. coli* strains of human origin. The emergence of resistance among the strains from animal and environmental origin has also been observed mainly due to the increased antibiotic usage in veterinary medicines, livestock management, and agricultural practices [13].

In the present research, similar resistance patterns have been observed in animal, human and environmental strains that indicate the firm interaction of all these ecologies. The environment could be a reservoir of antibiotic-resistant bacteria as environmental strains are exhibiting 14 distinct resistance patterns. This diverse antimicrobial resistance pattern in environmental strains may be due to the enhanced evolution under environmental stressors and/or interaction between the *E. coli* strains from human, animal, and environmental ecologies [14]. The antibiotic-resistant bacteria have been reported to be excreted from animals and humans in their feces that can interact with the environmental strains as animal and human fecal matter is being used as “manure” [15]. These strains may exchange antibiotic-resistant genes (ARG) via horizontal gene transfer leading to the diverse resistance patterns in environmental strains. The drug-resistant strains of human and animal origins may also be transferred to soil environments via wastewater as untreated wastewater/sewage water is used to irrigate crops in underdeveloped countries [16] like Pakistan [17]. Strains of human origin can acquire resistance patterns that are similar to that of animal strains either due to direct contact with the animals or by consuming animal products [18]. A study conducted in Mexico reported 92% resistance against ampicillin among *E. coli* strains isolated from beef and pork samples [19]. Furthermore, the diverse and medically important multi-drug resistant strains emerge due to the selection pressure, environmental contamination, or mixing that can be transferred to animals and humans through beach sand, food chain, recreational water, shellfish, and air [20].

The present investigation is providing evidence of the presence of strains with similar resistance patterns and virulence factors in animal, human and environmental niches. The genetic diversity of *E. coli* strains was evaluated by comparing ERIC-PCR-based fingerprints. ERIC constitutes the family of repetitive sequences in bacteria and is an excellent tool for bacterial fingerprinting [21]. ERIC-PCR fingerprinting is the preferred choice as it is as accurate as PFGE but simpler, time and cost-efficient, highly reproducible, and with great discriminatory power [22,23]. A total of 18 distinct genetic profiles were identified in all the 60 *E. coli* strains from human, animal, and environmental origins. The profile P1 was found shared among human, animal, and environmental strains indicating their genetic similarity and confirming the proposed mixing of human and animal origin strains with the environmental ones in soil and wastewater. The smaller number of sharing genetic profiles is attributed to the fact that the environmental strains cannot be directly compared with the ones extracted from living hosts due to differential survival rates in different ecologies [24]. Moreover, profile P4 was found common between animal and human strains indicating transmission between these two, most probably from animals to humans as these strains were resistant to macrolides and fluoroquinolones that are frequently used in livestock [25].

It is a very important feature of the microbial community in any niche to characterize microbial diversity. In the current study, richness, evenness, Shannon and Simpson’s diversity indices were calculated to estimate the *E. coli* genetic diversity in different ecological niches and found that soil was the most diverse ecological niche for the *E. coli* population followed by water with a maximum number of genetic profiles (6 each for soil and water) as compared to other sources. These results are also supporting our hypothesis that humans and animals are in direct contact with the soil thus making it a more diverse habitat and the soil itself can support the growth of *E. coli* strains and the genetic recombination that can increase the genetic diversity [26]. Microbes can enter the water bodies from the soil through different mechanisms including soil erosion and rainfall-mediated transport [27]. Moreover, the presence of pathogenic strains in soil and wastewater is further strengthening the proposed route of *E. coli* strains from animals and humans to the soil that is not the natural reservoir of pathogenic bacteria.

For pathotype determination, various genes have been amplified. It is important to note that 18/20 environmental strains are showing mixed pathotypes meaning that these are harboring all the studied genes. These results are revealing that various pathotypes have been transmitted from animals and humans to the environment where they got the opportunity to develop mixed pathotypes by gaining pathogenic genes most probably via horizontal gene transfer and then can enter back to human and animal ecologies. In the current study, it was observed that 18, 16, and 15 strains were positive for *eae*, *hylA*, and *stx*2, respectively, while no strain harbored the *stx1* gene out of a total of 20 human origin strains. Among the strains from animals, five and 18 were positive for *stx1* and *stx*2, respectively. These results were close to the study conducted in Shiraz, Iran, in which 146 STEC strains were isolated from 420 swab (rectal and mucosal) samples from cattle harboring the *stx*1 gene (15 isolates) and *stx*2 gene (78 isolates) [28]. In another study, a total of 514 STEC strains were isolated from diarrheic and healthy cows in Spain. PCR revealed that 278 isolates carried *stx*2 genes whereas 101 possessed *stx*1 genes [29]. The present study showed that 18/20 animal origin strains were positive for *eaeA* and 19/20 for *hlyA* gene, thus the prevalence was higher than the other results reported in the previous studies in different countries [30].

Five strains from the environmental source contained the *stx*1 gene, 18 were positive for both *stx*2 and *hlyA* whereas all the strains carried *eaeA* gene. A study in Egypt detected *stx*2 and *eae* genes in 98% and *stx*1 in 84% of *E. coli* O157 strains isolated from water samples [31]. Results of the virulence genes in the present study were supported by previous studies as discussed above, while variation was also observed which might be because of the sample selection like the source of the sample, sample type, and geographical area. High prevalence for the virulence genes *stx*2, *eaeA*, and *hlyA* was recorded in human, animal, and environmental strains in the current study. Isolates carrying these three virulence genes showed the highest resistance to ampicillin, ceftazidime, and gentamicin which was also observed in the previous study that strains that harbor these virulence genes were highly resistant to different antibiotics [32]. The high prevalence of the virulence genes is an alarming situation for human health and livestock because these virulence factors play a vital role in infections and enhances the pathogenicity of the microbes [33].

Moreover, the biofilm production capabilities of *E. coli* strains have been assessed as it is considered to protect bacteria from antibiotics and increase their pathogenic protection. In the current study, out of a total of 60 isolated strains, 13 were strong, 18 were moderate and 17 were weak biofilm producers. Resistance to more than seven antibiotics was detected in the strains with strong biofilm production. This observation is in agreement with a previous study that showed high resistance in sessile bacterial cells as compared to planktonic cells [34]. Biofilm-producing microscopic organisms are accountable for many intractable infections and are notoriously difficult to eliminate. They show resistance to antibiotics by several methods like restricted infiltration of antibiotics into biofilms, expression of resistance genes, and reduced growth rate [35].

The genetically diverse pathogenic multi-drug resistant *E. coli* strains are disseminated throughout the animal, human and environmental ecologies that could be clinically important and a major threat to public health. The study revealed that drug-resistant pathogenic *E. coli* strains could be transmitted bi-directionally among the environment, humans, and animals. These strains enter the environment (soil and water) majorly through the application of manure and the genetically diverse, antimicrobial resistant, and virulent strains could emerge that can infect animals and humans. The use of antibiotics in animals and aquaculture should be limited and manure should be investigated for bacterial diversity and concentration before being used as fertilizer. The release of wastes from pharmaceutical companies and other industries into the environment without proper treatment should be strictly inhibited. Moreover, this is the need of the time that the antibiotic resistance analysis should be made under the umbrella of one health framework to facilitate information sharing between investigators and decision-makers at all levels.

## 4. Materials and Methods

### 4.1. Sample Collection and Processing

A total of 156 samples (42 from environment, 40 from animals and 74 from humans) were collected under sterile conditions. Soil samples were collected from five different agricultural sites (Table S4) at the depth of 5–10 cm to avoid dead cells. Wastewater samples were collected from seven different water bodies receiving sewage and industrial wastes. Animal samples were collected from five different farms and human samples were collected from various tertiary care hospitals of Pakistan including The University of Lahore Teaching Hospital (Lahore), Sharif City Complex (Lahore), Jinnah Hospital (Lahore), Rehman Medical Complex (Peshawar), Nishtar Hospital (Multan) and District Head Quarter Hospital (Rawalpindi). Suspensions of soil samples (w/v) and wastewater (v/v) samples were prepared using 0.1 mL of sterile saline and cultured on MacConkey agar. Samples other than soil and wastewater were streaked on MacConkey agar and were incubated at 35 °C for 24 h. After incubation, *E. coli*, which were characterized as pink, smooth, and rounded colonies were differentiated by Gram’s reaction. The isolates that showed positive results for methyl-red and indole tests while negative for H_2_S production were selected for further analysis.

### 4.2. Biochemical and Molecular Characterization of E. coli

Analytical profile index (API) 20E system (Biomerieux, Lyon, France) was used to confirm the identification of *E. coli* isolates. DNA was extracted with a High Pure Viral Nucleic Acid Kit (Roche Cat. No. 11 858 874 001) according to the manufacturer’s instructions. For molecular characterization, the uidA gene, responsible for the synthesis of beta-glucuronidase enzyme and intrinsic in all *E. coli* strains, was amplified by PCR (Table S5). PCR reaction mixture was carried out in a 20 µL volume containing two µL of the nucleic acid template (approximately 60 ng of DNA), 0.75 µL of each primer, and 10 µL master mix (Phusion flash PCR). *E. coli* ATCC 43890 was used as a positive control, while deionized distilled water was used as a negative control. DNA ladder (Gene ruler 1 kb by Thermo scientific, Boston, MA, USA) was run to estimate the size of PCR products.

### 4.3. Antibiotic Resistance Pattern

Resistance pattern of *E. coli* against various antimicrobial agents (Oxoid, Basingstoke, UK) including carbapenems (imipenem (IMP), meropenem (MEM)), Penicillins (ampicillin (AM)), Cephalosporins (cefotaxime (CTX), ceftazidime (CAZ)), Aminoglycosides (amikacin (AK), gentamicin (CN)), Fluoroquinolones (ciprofloxacin (CIP), levofloxacin (LEV)), Sulfonamides (trimethoprim-sulfamethoxazole (SXT)), Tetracyclines (tetracycline (TE)), Monobactams (aztreonam (AZT)), and Macrolides (azithromycin (AZM)) was determined on Mueller Hinton (MH) agar plates by Kirby Bauer disc diffusion method to declare whether the organism is multidrug-resistant (MDR), extensive drug-resistant (XDR) and/or pan drug-resistant (PDR). The MIC of tigecycline (TGC) was estimated by the broth microdilution method and interpreted as per FDA criteria [36]. The microbe which is resistant to at least one drug in three or greater than three antibiotic categories was termed as MDR while the organism which is resistant to at least 1 agent in all but two and fewer antibiotic classes was regarded as XDR and the organism resistant to all available antibiotic classes was declared as PDR [37]. Results were interpreted according to clinical & laboratory standard institute (CLSI) guidelines.

### 4.4. Detection of Biofilm Formation

The tissue culture plate test is considered a gold-standard method for biofilm detection. *E. coli* isolates were inoculated into 10% trypticase soy broth (TSB) and incubated for 18 h at 37 °C. The stationary-phase cultures were vortexed and diluted at 1:1000 in a TSB medium supplemented with one percent glucose. Each diluted culture (200 µL) was loaded into a separate well of 96 wells of a flat-bottom microtiter plate (Sigma Aldrich, Burlington, MA, USA). The plate was covered and incubated for 24 h at 37 °C. After incubation, the contents of the plate were discarded, and wells were washed thrice with pre-warm phosphate-buffered saline (pH 7.2). The biofilm in each well was fixed with two percent sodium acetate and stained with 0.1% crystal violet for 20 min. The excess stain was washed with double distilled and deionized water and the plate was air-dried. Optical density (OD) was taken at 570 nm by using an ELISA plate reader.

### 4.5. Detection of Virulence Genes

Four virulence genes namely *stx*1, *stx*2, *hlyA*, and *eaeA* were amplified by using specific primer sets (Table S5). PCR reaction mixture was carried out in a 20 µL volume containing two µL of the nucleic acid template (approximately 60 ng of DNA), 0.75 µL of each primer set, and 10 µL master mix (Phusion flash PCR). *E. coli* ATCC 43890, ATCC 43889, and ATCC 43887 reference strains were used as a positive control for *stx*1, *stx*2, and *eaeA*, *hlyA*, respectively, while distilled water was used as a negative control. Thirty cycles were run, and each cycle was consisted of denaturation at 95 °C for three sec, annealing at 62 °C for 10 s and extension at 72 °C for 15 s with the final extension at 72 °C for 10 min. The PCR products were resolved on one percent agarose gel at 70 V for 40 min and visualized on Gel Doc EZ System (Bio-Rad, Hercules, CA, USA, 1708270).

### 4.6. Enterobacterial Repetitive Intergenic Consensus (ERIC-PCR)

Genetic diversity among *E. coli* from various sources was established by ERIC-PCR using a specific set of primers (ERIC-1; 5′-ATGTAAGCTCCTGGGGATTCA-3′ and ERIC-2; 5′-AAGTAAGTGACTGGGGTGAGCG-3′). PCR reaction mixture was carried out in 20 µL volume containing two µL bacterial DNA, one µL primer, 10 µL master mix, and seven µL deionized distilled water. The PCR was carried at 94 °C for three sec, 52 °C for 10 s and 72 °C for two min with an initial denaturation at 94 °C for 10 s and final extension at 72 °C for 5 min.

### 4.7. E. coli Genetic Diversity Evaluation

*E. coli* genetic diversity based on unique ERIC genomic profiles was calculated for different origins and sources using the Shannon-Wiener diversity index $\left[H=-\sum_{i=1}^{S}\left(Pi\right)\left(logPi\right)\right]$ and Simpson’s diversity index $\left[1-D=-\sum_{i=1}^{S}(Pi^{2})\right]$, where *S* and *Pi* represent the total number of unique profiles and number of organisms with unique profile (n)/total number of organisms in the community (N), respectively. PAST software (V. 4.03) was used to calculate these indices along with richness and evenness.

### 4.8. Statistical Analysis

Data were tabulated and evaluated with the help of SPSS (V25.0). Percentage (%) was used for categorical data. Frequency was calculated for qualitative variables. The Chi-square test was employed to calculate the association between two parameters. *p*-value ≤ 0.05 was taken as statistically significant. Bionumerics (V 7.6) was used to construct dendrograms based on Dice similarity with 1% band tolerance and UPGMA (unweighted pair group method using average linkages). PAST (V 4.03) was employed to calculate diversity indices.

## Figures and Tables

**Table 1:** Profiling of *E. coli* strains based on multiple antibiotic resistance patterns.

Resistance Profiles	Antibiotic Combinations	Number of *E. coli* Strains (*n*)		
		Human Origin	Animal Origin	Environmental Origin
R1	Penicillin/Cephalosporins/Sulfonamides/ Tetracycline/Aminoglycosides/Monobactams	2	2	1
R2	Penicillin/Cephalosporins/Fluoroquinolones/ Sulfonamides/Tetracycline/Aminoglycosides	8	-	1
R3	Cephalosporins/Sulfonamides/Tetracycline/ Aminoglycoside/Monobactams	-	-	1
R4	Cephalosporins/Fluoroquinolones/ Tetracycline/Aminoglycosides	-	-	1
R5	Penicillin/Fluoroquinolones/Sulfonamides/ Tetracycline/Carbapenems/Monobactams/ Macrolides	-	-	1
R6	Penicillin/Cephalosporins/Sulfonamides/ Aminoglycosides	1	-	1
R7	Penicillin/Cephalosporins/Fluoroquinolones/ Sulfonamides/Tetracycline	1	-	-
R8	Penicillin/Cephalosporins/Fluoroquinolones/ Sulfonamides/Monobactams	1	-	-
R9	Penicillin/Cephalosporins/Fluoroquinolones/ Sulfonamides/Tetracycline/Aminoglycosides/ Carbapenems	5	-	-
R10	Penicillin/Cephalosporins/Sulfonamides/ Tetracycline/Monobactams/Macrolides	1	-	-
R11	Penicillin/Cephalosporins/Sulfonamides/ Tetracycline/Aminoglycosides	1	5	2
R12	Penicillin/Cephalosporins/Tetracycline/ Aminoglycosides	-	5	5
R13	Penicillin/Cephalosporins/Fluoroquinolones/ Sulfonamides/Tetracycline/Aminoglycosides/ Carbapenems/Macrolides	-	2	-
R14	Penicillin/Cephalosporins/Fluoroquinolones/ Tetracycline/Monobactams/Macrolides	-	1	1
R15	Penicillin/Cephalosporins/Fluoroquinolones/ Aminoglycosides	-	1	1
R16	Penicillin/Cephalosporins/Aminoglycosides	-	4	2
R17	Penicillin/Cephalosporins/Fluoroquinolones/ Monobactams	-	-	1
R18	Penicillin/Cephalosporins/Fluoroquinolones/ Tetracycline/Aminoglycosides/Carbapenems	-	-	1
R19	Penicillin/Cephalosporins/Tetracycline/ Aminoglycosides/Carbapenems	-	-	1

**Table 2:** Prevalence of virulence genes of human, animal, and environmental strains.

Virulence Genes	*E. coli* Strains			
	Human (*n* = 20)	Animal (*n* = 20)	Environment (*n* = 20)	Total (*n* = 60)
*stx1*	0	5	5	10
*stx2*	15	18	18	51
*eaeA*	18	18	20	56
*hlyA*	16	19	18	53

**Table 3:** Association of biofilm production with antibiotic resistance.

Antibiotic Groups	Biofilm			
	Non-Producer		Producer	
	Resistant *n* (%)	Sensitive *n* (%)	Resistant *n* (%)	Sensitive *n* (%)
Penicillins	11 (92)	01 (8)	47 (98)	1 (2)
Cephalosporins	12 (100)	0 (0)	47 (98)	1(2)
Fluoroquinolones	7 (58)	5 (42)	28 (58)	20 (42)
Sulfonamides	4 (33)	8 (67)	23 (48)	25 (52)
Tetracycline	7 (58)	5 (42)	41 (85)	7 (15)
Aminoglycosides	10 (83)	2 (17)	43 (89)	5 (10)
Carbapenems	0 (0)	12 (100)	10 (21)	38 (79)
Monobactams	2 (17)	10 (83)	10 (21)	38 (79)
Macrolides	1 (8)	11 (92)	5 (10)	43 (89)

**Table 4:** Diversity indices values for the human, animal, and environmental *E. coli* strains.

Origins and Sources	Shannon (H)	Simpson (1-D)	Evenness (E)	Richness (S)
Human	1.7	0.7	0.6	9.0
Urine	1.5	0.7	0.9	5.0
Pus	0.5	0.3	0.8	2.0
Sputum	0.9	0.5	0.8	3.0
Blood Culture	0.6	0.5	1.0	2.0
Tissue culture	0.6	0.5	1.0	2.0
Animal	1.8	0.7	0.7	8.0
Cattle Faeces	1.6	0.7	0.8	6.0
Cattle Intestine	1.2	0.6	0.8	4.0
Cattle Milk	1.3	0.7	0.9	4.0
Environment	1.7	0.8	0.9	6.0
Wastewater	1.7	0.8	0.9	6.0
Soil	1.7	0.8	0.9	6.0

## Data Availability

Not applicable.

## Supplementary Materials

The following supporting information can be downloaded at: https://www.mdpi.com/article/10.3390/antibiotics11081061/s1, Table S1: Antimicrobial susceptibility (S) and resistance (R) against *E. coli* strains of Humans, Animals and Environmental origins. Table S2: Biofilm and non-biofilm producer strains of human, animal and environmental origin. Table S3: ERIC-PCR based fingerprints of genetic profiles. Table S4: Geographical locations of sampling sites. Table S5: Primer sets used in the study. Figure S1: Clustering of *E. coli* strains from human (a), animal (b) and environmental (c) origins based on antimicrobial susceptibility patterns. Figure S2. Heatmap showing the virulence genes profiles of all *E. coli* strains. Figure S3: Distribution of *E. coli* pathotypes among human, animal and environmental origins and sources. Figure S4: Association of pathotypes with antimicrobial resistance profiles (*p*-value 0.08). Figure S5: ERIC-PCR fingerprints based dendrograms of human (a), animal (b), environmental (c) strains based on Dice similarity coefficient UPGMA.
