# Supplementary material for: Genetic Diversity and Virulence Profiling of Multi-Drug Resistant Escherichia coli of Human, Animal, and Environmental Origins
Source: Antibiotics (Basel). 2022 Aug 4;11(8):1061. doi: 10.3390/antibiotics11081061 (PMC9405421; doi:10.3390/antibiotics11081061)
Supplement: Supplementary file 1 [file antibiotics-11-01061-s001.zip › antibiotics-1791252-supplementary.pdf]

Table S1: Antimicrobial susceptibility (S) and resistance (R) against *E. coli* strains of Humans, Animals and Environmental origins.

Drugs	Breakpoints (mm)		Samples isolated					
			Humans (n=20)		Animals (n=20)		Environmental (n=20)	
	S	R	R	S	R	S	R	S
**CIP**	≤ 21	≥ 26	n=17<br>85%	n=03<br>15%	n=09<br>45%	n=11<br>55%	n=08<br>40%	n=12<br>60%
**LEV**	≤ 16	≥ 21	n=17<br>85%	n=03<br>15%	n=07<br>35%	n=13<br>65%	n=09<br>45%	n=11<br>55%
**CTX**	≤ 22	≥ 26	n=18<br>90%	n=02<br>10%	n=15<br>75%	n=05<br>25%	n=13<br>65%	n=07<br>35%
**CAZ**	≤ 17	≥ 21	n=20<br>100%	n=00<br>00%	n=18<br>90%	n=02<br>10%	n=17<br>85%	n=03<br>15%
**CN**	≤ 12	≥ 15	n=16<br>80%	n=04<br>20%	n=17<br>85%	n=03<br>15%	n=15<br>75%	n=05<br>25%
**AK**	≤ 14	≥ 17	n=03<br>15%	n=17<br>85%	n=09<br>45%	n=11<br>55%	n=07<br>35%	n=13<br>65%
**TGC**	≤ 15	≥ 18	n=09<br>45%	n=11<br>55%	n=14<br>70%	n=06<br>30%	n=08<br>40%	n=12<br>60%
**TE**	≤ 11	≥ 15	n=14<br>70%	n=06<br>30%	n=05<br>25%	n=15<br>75%	n=08<br>40%	n=12<br>60%
**IMP**	≤ 19	≥ 23	n=05<br>25%	n=15<br>75%	n=02<br>10%	n=18<br>90%	n=02<br>10%	n=18<br>90%
**MEM**	≤ 19	≥ 23	n=05<br>25%	n=15<br>75%	n=02<br>10%	n=18<br>90%	n=02<br>10%	n=18<br>90%
**AMP**	≤ 13	≥ 17	n=20	n=00	n=18	n=02	n=17	n=03

			100%	00%	90%	10%	85%	15%
**SXT**	≤ 10	≥ 16	n=18<br>90%	n=02<br>10%	n=01<br>05%	n=19<br>95%	n=05<br>25%	n=15<br>75%
**AZT**	≤12	>12	n=4<br>20%	n=16<br>80%	n=3<br>15%	n=17<br>85%	n=5<br>25%	n=15<br>75%
**AZM**	≤ 4	≥ 16	n=1<br>5%	n=19<br>95%	n=3<br>15%	n=17<br>85%	n=2<br>10%	n=18<br>90%

CIP=ciprofloxacin, LEV=levofloxacin, CTX=cefotaxime, CAZ=ceftazidime, CN=gentamicin, AK=amikacin, TGC=tigecycline, TE=tetracycline, IMP=imipenem, MEM=meropenem, AM=ampicillin, SXT=trimethoprim-sulfamethoxazole, AZT=aztreonam, AZM= Azithromycin

Table S2: Biofilm and non-biofilm producer strains of human, animal and environmental origin.

Biofilm production	*E. coli* strains			
	Human (n=20)	Animal (n=20)	Environment (n=20)	Total (n=20)
**Strong positive**	05	04	04	13 (21.6%)
**Moderate positive**	07	06	05	18 (30.0%)
**Weak positive**	03	06	08	17 (28.3%)
**Non biofilm producers**	05	04	03	12 (20.0%)

Table S3: ERIC-PCR based fingerprints of genetic profiles.

Band Size (bp)	Genomic Profiles																	
	P1	P2	P3	P4	P5	P6	P7	P8	P9	P10	P11	P12	P13	P14	P15	P16	P17	P18
6000												+						
4500				+														
4000													+					
3800			+															
3500														+				
2900	+									+		+	+		+			
2400							+											
1700			+								+	+			+			
1600	+	+		+					+	+			+			+		
1500					+													
1400							+	+	+	+	+	+	+					
1300					+													
1200				+		+	+		+									
1100	+	+	+	+	+		+	+		+	+		+	+	+	+	+	+

850	+	+						+			+			+	+			+
750				+	+		+	+		+		+	+	+	+	+	+	
650											+							
400				+	+		+	+		+								+
350											+							
250					+		+											

Table S4: Geographical locations of sampling sites

Sources	No. of Isolates (n=60)	Geographical Distribution of Sampling Sites
Wastewater	13	Kala Shah Kaku<br>Sundar industrial estate,<br>Kasur,<br>College Road,<br>Valencia town,<br>Thokar Niaz Baig<br>Ada plot
Soil	07	Bedian Road,<br>Manga mandi,<br>Raiwind,<br>Ravi interchange<br>Kasur
Cattle faeces	09	Animal samples were collected from 5 different farms, Johar town block F,<br>Edwn lane villas 2,<br>Khna nau kahna,<br>Valencia town and<br>Sarwar
Rectal swab	06	
Milk	05	
Urine	06	Uol Teaching hospital, Lahore<br>Sharif city complex, Lahore<br>Jinnah hospital, Lahore<br>Rehman Medical complex, Peshawer<br>Nishter hospital, Multan<br>District Head Quarter Hospital, Rawalpindi
Pus	05	
Sputum	05	
Blood culture	02	
Tissue culture	02	

Table S5: Primer sets used in the study

Primers	Primer Type	Primer sequences (5'→3')	Annealing Temperature (°C)	Product Size (bp)
uidA	Forward<br>Reverse	TGGTAATTACCGACGAAAACGGC<br>ACGCGTGGTTACAGTCTTGCG	57	162
hlyA	Forward<br>Reverse	GCATCATCAAGCGTACGTTCC<br>AATGAGCCAAGCTGGTTAAGCT	60	534
Stx1	Forward<br>Reverse	ACACTGGATGATCTCAGTGG<br>CTGAATCCCCCTCCATTATG	58	614
Stx2	Forward<br>Reverse	CCATGACAACGGACAGCAGTT<br>CCTGTCAACTGAGCAGCACTTTG	56	779
eaeA	Forward<br>Reverse	GACCCGGCACAAGCATAAGC<br>CCACCTGCAGCAACAAGAGG	58	384

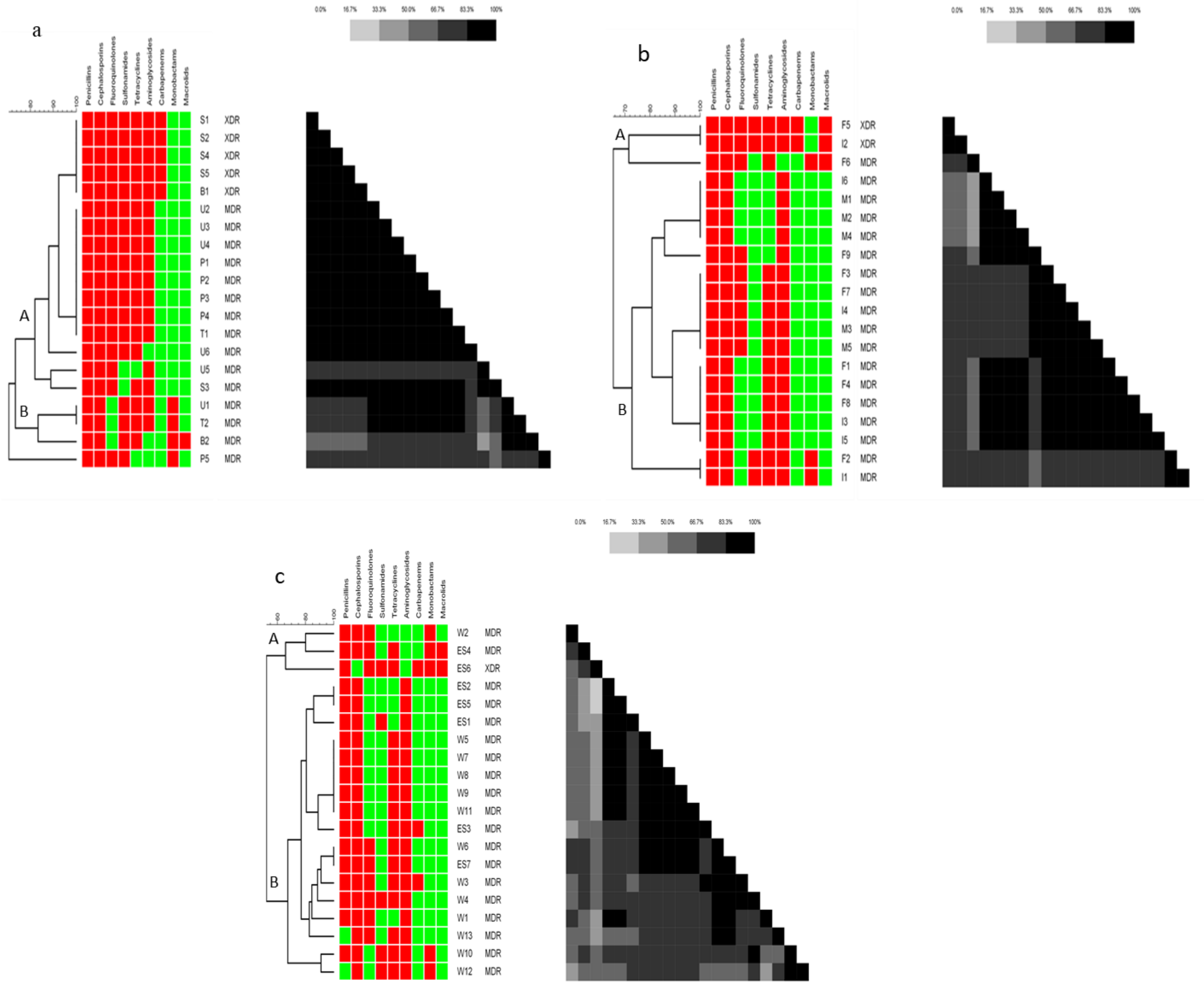

Figure S1: Clustering of *E. coli* strains from human (a), animal (b) and environmental (c) origins based on antimicrobial susceptibility patterns.

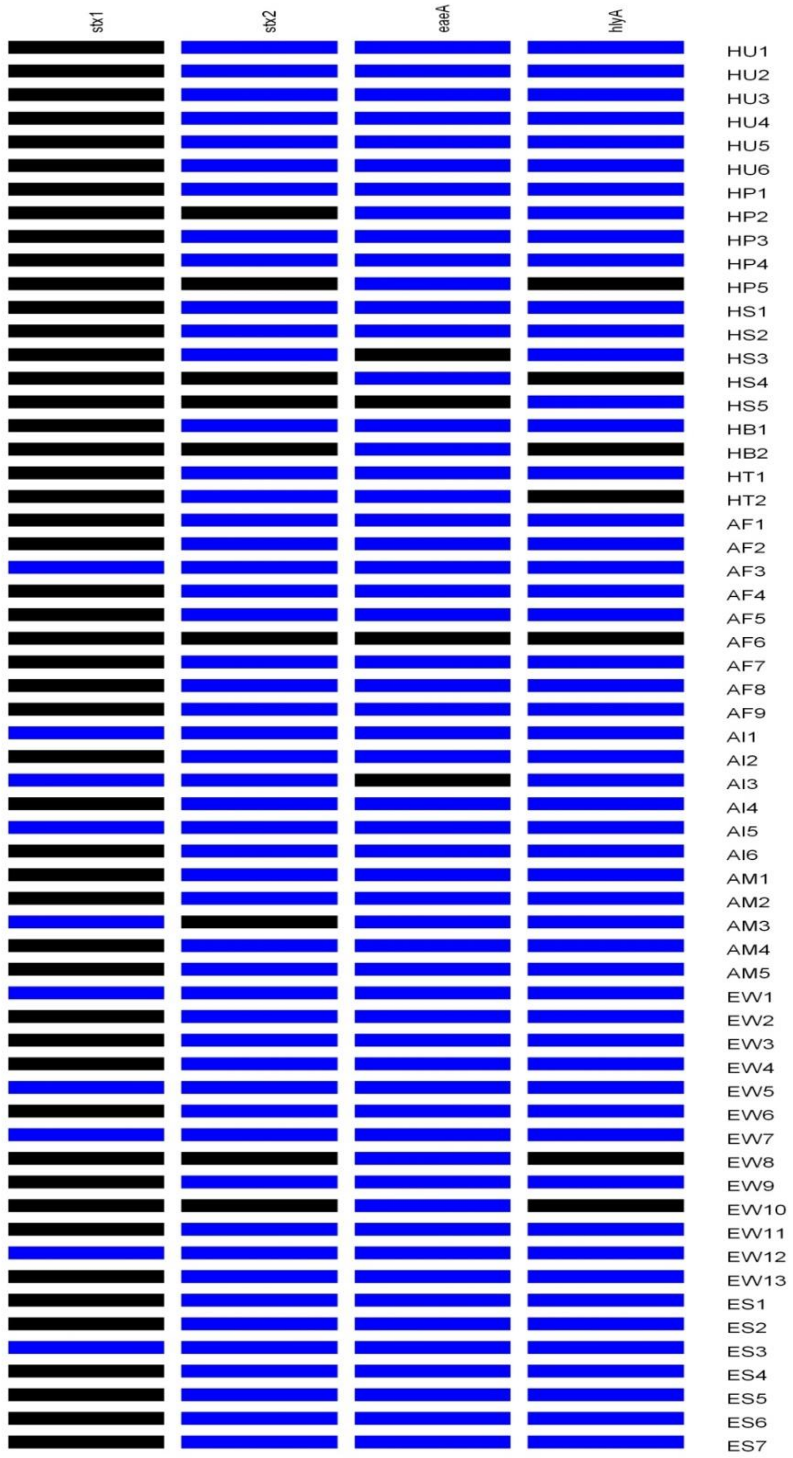

Figure S2. Heatmap showing the virulence genes profiles of all *E. coli* strains. Blue colour indicates the presence and while black colour indicates the absence of the gene. E letter is used for environment, A for animal and H for human origin.

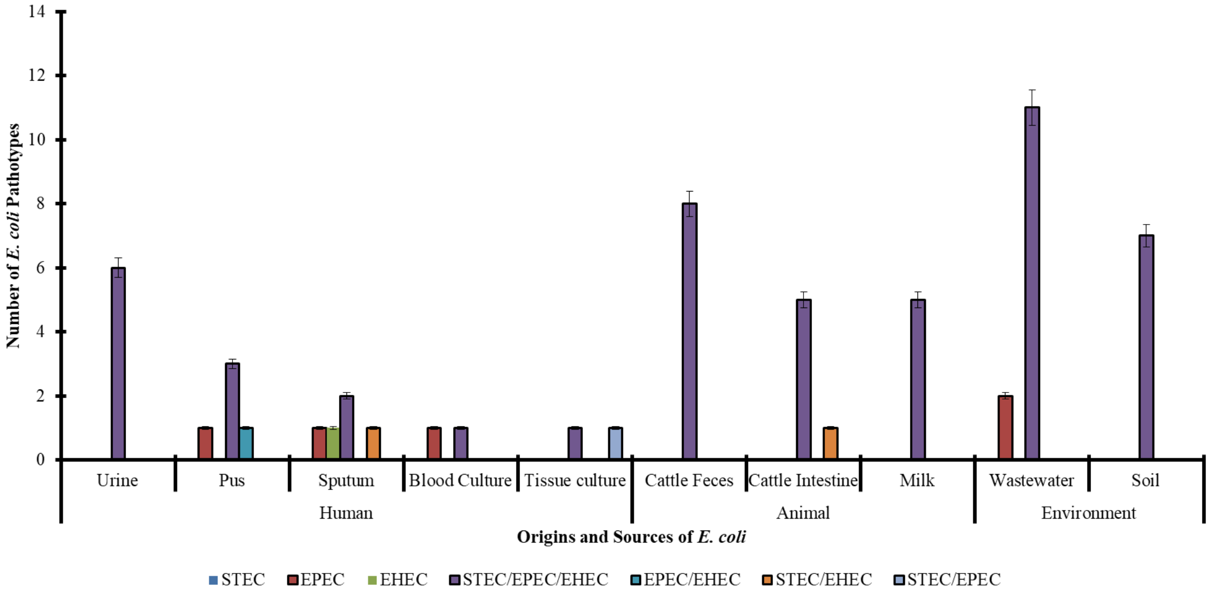

Figure S3: Distribution of *E. coli* pathotypes among human, animal and environmental origins and sources.

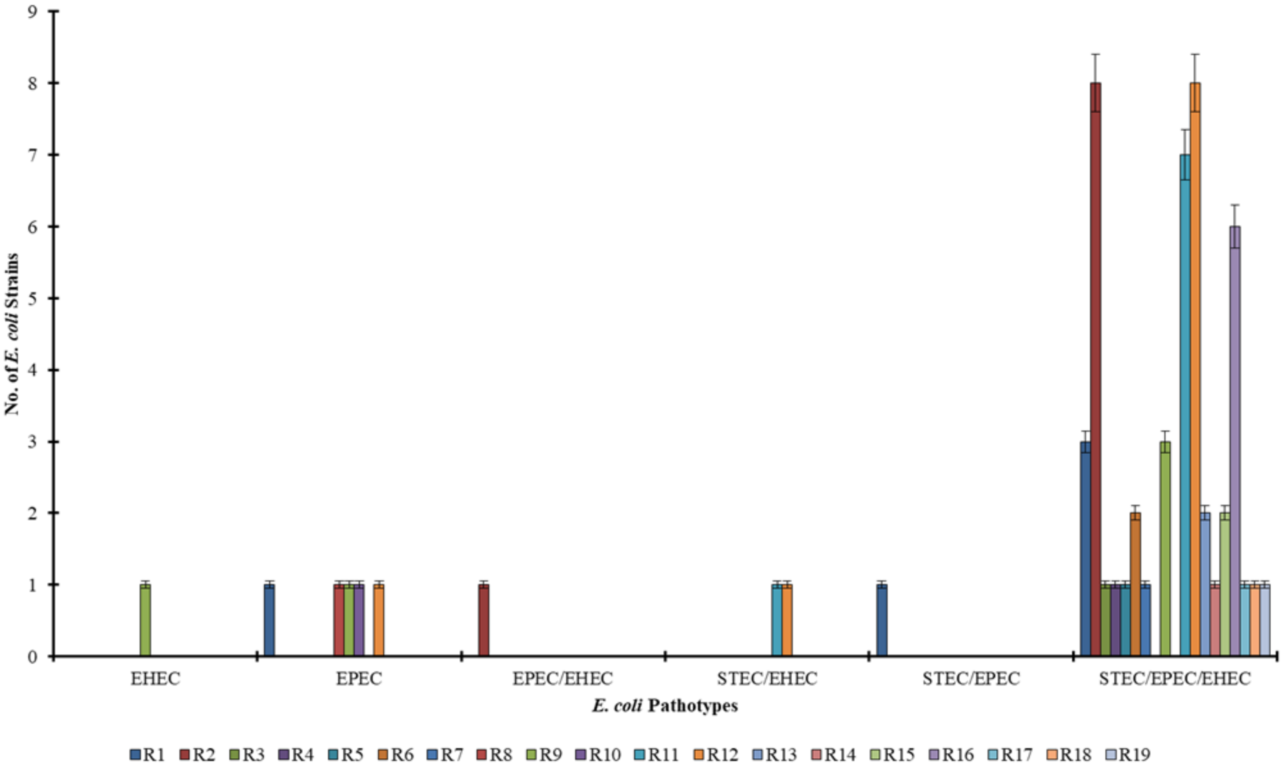

Figure S4: Association of pathotypes with antimicrobial resistance profiles (*P*-value 0.08).

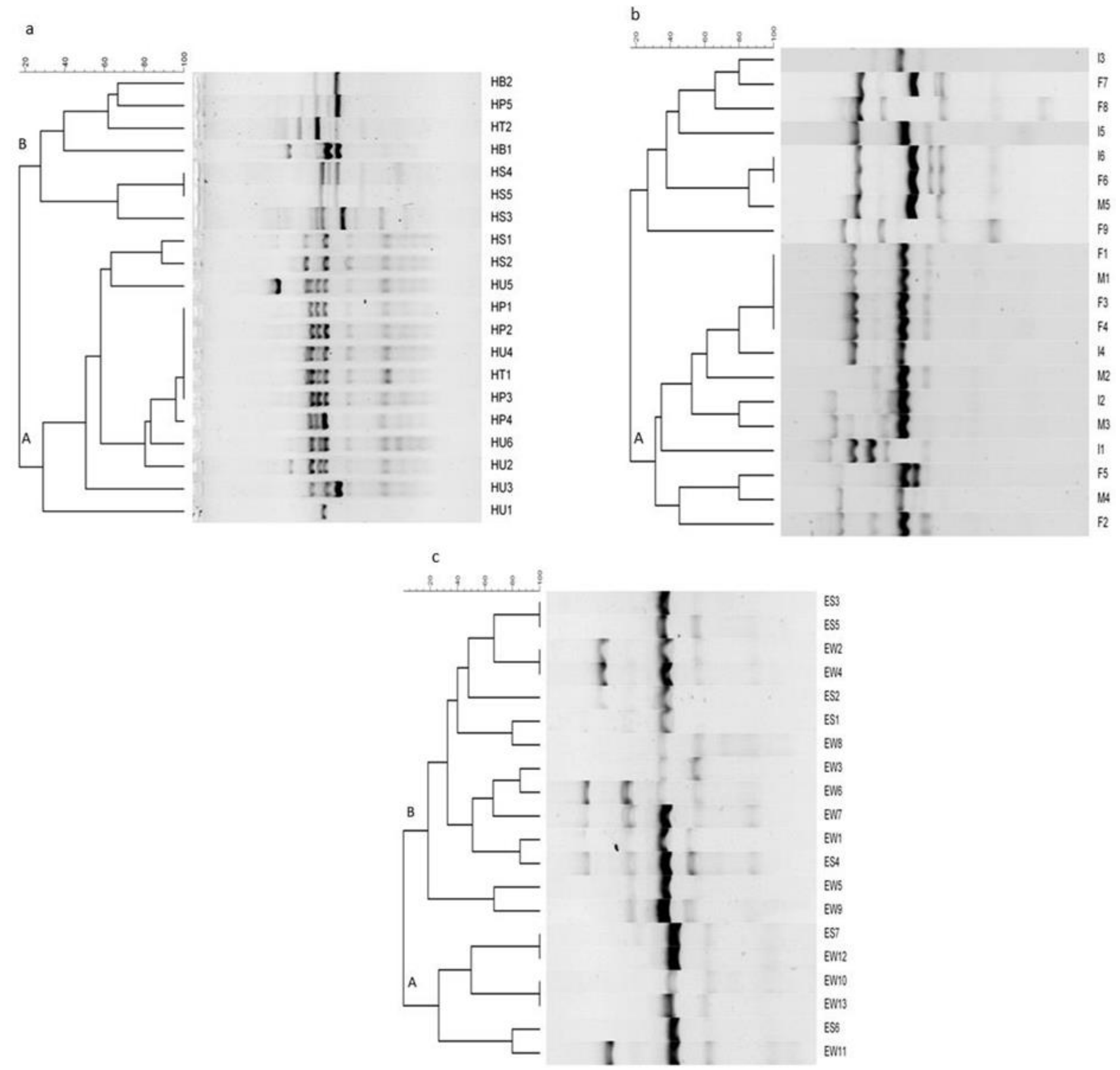

Figure S5: ERIC-PCR fingerprints based dendrograms of human (a), animal (b), environmental (c) strains based on Dice similarity coefficient UPGMA.
